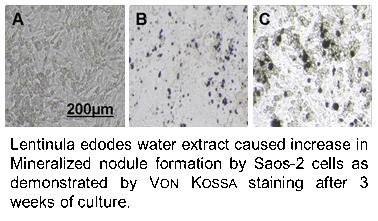# In vitro bone inducing effects of *Lentinula edodes* (shiitake) water extract on human osteoblastic cell cultures

**DOI:** 10.1007/s13659-013-0070-3

**Published:** 2013-12-03

**Authors:** Ashraf Saif, Kristian Wende, Ulrike Lindequist

**Affiliations:** 1 Al-Leith University College, Umm Al-Qura University, Makkah, 311 Saudi Arabia; 2 Institute of Pharmacy, Ernst-Moritz-Arndt-University Greifswald, Friedrich-Ludwig-Jahn-Straße 17, D-17489 Greifswald, Germany

**Keywords:** medicinal mushrooms, *Lentinula edodes*, osteoporosis, alkaline phosphatase, bone mineralization, shiitake

## Abstract

The effect of *Lentinula edodes* water extract (LE) on two osteoblastic cell cultures (HOS 58 and Saos-2) was investigated to determine if this edible medicinal mushroom has osteoinductive properties. Activity of alkaline phosphatase and mineralization were used as indicators for the vitality and maturation of the bone cells. Cultivation of human osteosarcoma cells HOS 58 for five days in presence of a serial dilution of the aqueous extract of *L. edodes* (0.8 μg/mL-125 μg/mL) resulted in a significant elevation of alkaline phosphatase activity (ALP) of the cells in comparison to untreated cells. Saos-2 cells, incubated with LE (20 μg/mL) and *β*-glycerol phosphate (2 mM) for 21 days, displayed a 2 fold level of mineralization than cells cultured soley with the positive control, β-glycerophosphate. The obtained results clearly indicate the activity of LE as a bone inducing agent *in vitro*. Therefore, the shiitake mushroom (*L. edodes*) deserves attention as a supportive dietary treatment or nutraceutical in the case of diseases accompanied with bone disorder, such as osteoporosis, osteopenia, and late complication of diabetes.

## Introduction

Postmenopausal osteoporosis is a major age-related health problem for women. The cause may be due to negative calcium balance due to dietary insufficiency, or decreased intestinal calcium absorption, as well as increase in urinary calcium loss associated with estrogen deficiency during menopause[1]. Hormone replacement therapy (HRT) has been an established regime for the prevention of post-menopausal bone loss[2,3]. Recent evidence indicates that its long-term use is accompanied by side effects, such as the increased risk of breast, ovarian and endometrial cancer[4,5]. Thus, complementary dietary treatment with established efficacy and safety should be developed for the prevention and treatment of post-menopausal osteoporosis.

Traditional Chinese Medicine (TCM) has been widely used for thousands of years to treat fractures and joint diseases. There are a multitude of herbs that are used in TCM formulas for the prevention and treatment of osteoporosis[6]. Although these herbal medicines are seen as cost-effective alternatives by their traditional users, their international acceptance as a major regimen for prevention and/or the treatment of osteoporosis, would require extensive research using modern scientific methods of therapeutic validation.

*To whom correspondence should be addressed. E-mail: aaa_saif@hotmail.com

Mushrooms are defined as the fruiting body of a macrofungus. Further, mushrooms contain an array of vitamins; particularly niacin, thiamine, riboflavin, biotin and vitamin C. In addition, mushrooms contain a wide variety of bioactive molecules including terpenoids, steroids, phenols, nucleotides, glycoproteins and polysaccharides[7]. Certain Mushrooms are claimed in traditional medicine to exhibit anti-tumor, antiviral, antibiotic, anti-inflammatory, hypoglycemic, hypocholesterolemic and hypertensive activities as well as having therapeutic and nutritional value[8–10]. One in particular is *Grifola frondosa*, a widely used mushroom in TCM and Kampo. It is indicated to have various actions such as an anti-diabetic and an adjuvant antitumor activity and was proved before by us to have anti-osteoporosis effect[11]. In the course of our screening investigations for interesting biological activities and novel compounds in medicinal mushrooms[12] we identified *L. edodes* as further mushroom with stimulating effects on bone cells.

Shiitake is the common Japanese name for the edible mushroom *Lentinula edodes*[13], which is now cultivated and is the second most commonly produced edible mushroom in the world[14]. It grows naturally on fallen wood of broadleaf forests. A Chinese physician of the Ming Dynasty (1368–1644), Wu Juei, wrote extensively about this mushroom and noted its ability to increase energy, cure colds, eliminate intestinal worms, preserve and promote health and to improve circulation and metabolism[15]. Today, *L. edodes* is used in the traditional medicine as an immune modulator for improvement of life quality and for prevention of metabolic diseases. Lentinan, a

*β*-glucan isolated from the mushroom, is used in evidence-based medicine for adjuvant tumor therapy[10,15].

*Lentinula edodes* has been shown to contain a hypocholesteremic compound originally named "lentysine" or "lentinacin" but it was later isolated and identified as eritadenine[16]. It was observed that *L. edodes* reduced blood serum cholesterol levels in rats fed a 1% cholesterol diet[16,17]. Moreover, it was revealed that a "neutral detergent fiber" (NDF) extracted from *L. edodes* also displayed a cholesterol-lowering effect distinct from that of eritadenine[18]. 'Statins', are natural occurring or synthetic cholesterol lowering agents and have been shown to stimulate bone formation in laboratory studies, both *in vitro* and *in vivo*[19–22]. Due to the correlation between cholesterol lipid-lowering drugs and osteoporosis, we presume that a nutraceutical which exerts cholesterol lowering effects may also show protectant effects against osteoporosis (osteoinductive effect). The presented study was conducted to show evidence of *in vitro* osteoinductive effect, to extend our bioprospecting of this medicinal mushroom and to confirm whether or not *Lentinula edodes* extract (LE) exerts osteoinductive effects on osteoblastic cell *in vitro*. Before, only UV irradiated samples of *L. edodes* with an increased content of Vitamin D2 and added calcium have been investigated for their osteoporosis-related effects in mice[23].

## Results and Discussion

Due to the established hypocholesterolemic effect of *Lentinula edodes* (LE) and the possible relationship between plasma cholesterol concentration and osteoporosis via cholesterol synthesis and bone-resorbing osteoclast cells activation use of the same pathway[24], we assumed that LE would modulate bone cells as well. Thus we studied the influence of an aqueous extract of this mushroom on growth and maturation of human osteoblastic osteosarcoma cell lines; HOS 58 and Saos-2. A detailed analysis of the myco-chemical composition of LE was not conducted but rather a whole water extract was investigated. Initially, we performed HPLC fingerprinting using RP18 separation to determine the myco-chemical profile (see Figure 1). A majority of the components are only weakly retained on the column and were eluted rapidly (no obvious peaks after 9 minutes of retention time). Moreover, UV absorption was rather weak, with most constituents being detected solely at 206 nm absorption, indicating the low presence of conjugated double bounds. This observation was expected for a water soluble extract and is indicative for the presence of carbohydrate compounds. This was confirmed by TLC (data not shown).

Osteoblastic-mediated bone formation can be divided into three phases: proliferation, matrix maturation, and mineralization[25]. This complex process can only in part be reflected using cell lines *in vitro*[26]. However, in order to increase the validity of our results and to impede any wrong-positive data we used two different cell lines. In particular, the Saos-2 cell line is widely recognized as an *in vitro* bone model. It features many typical properties of *in vivo* osteoblasts, such as cytokine production and also matrix mineralization, the hall mark of osteoblasticity. Moreover, HOS 58 cells were found to be suitable for *in vitro* screening because of their sensitive alkaline phosphatase expression[27]. We used cell growth as an indicator for the proliferation phase, alkaline phosphatase activity as parameter for matrix maturation and lastly mineralization of the extracellular matrix (ECM).

**Cell Vitality.** Tetrazolium-based colorimetric assays are one of the most common methods to detect mammalian cell survival and proliferation. MTT is a yellow water-soluble tetrazolium dye which is reduced to a purple water-insoluble formazan by living cells[29,30]. The amount of formazan generated is proportional to the number of viable cells.

Cytotoxicity testing of LE did not display any cytotoxicity as measured via the reduction of cell viability (Figure 2). Even at the highest concentration tested (120 µg/mL) HOS 58 cells

**Figure 1.** Structures HPLC fingerprint of *L. edodes* extract (30 µg on column), RP18 water/formic acid – methanol gradient. Black = 206 nm, red = 254 nm, small inset shows enlarged 254 nm detection.

**Figure 2.** Effect of different concentrations of *Lentinula edodes* total water extract on proliferation of HOS 58 osteosarcoma cells was assessed by MTT assay. No significant differences were observed within the concentration range tested. Each bar represents mean ± SD (n = 24) and was generated by three independent experiment.

maintained their metabolic activity. We did not observe an increase in cell number or viability at any concentration tested, indicating that LE has no pro-mitotic effects.

**Influence on Total Cellular Protein Content.** Data presented in Figure 3 show that LE extract in concentrations until 30 µg/mL did not influence protein production by HOS 58 cells. Above that concentration protein production reduces, reaching only 85% of the vehicle control. Due to the lack of cytotoxicity, this can only be considered as a stimulation of cell maturation. The latter would lead to a reduction of ECM production and thus lower protein content of the cell (and matrix) lysate. We did not observe an increase of cellular protein at any concentration tested. These observations support the hypothesis that LE promotes cell maturation, reduces cell proliferation and extracellular matrix (ECM) production, respectively.

**Figure 3.** The effect of different concentrations of *Lentinula edodes* total water extract on total cellular protein of HOS 58 cells. Protein content was calculated via standard curve using BSA. Each bar represents mean ± SD (n = 24) and was generated by three independent experiments.

**Influence on Activity of Alkaline Phosphatase.** Alkaline phosphatase (ALP) activity is commonly used as an indicator of osteoblastic cell maturation. The enzyme is considered to mark the middle stage of bone formation and further appears during the matrix maturation phase. Currently, the enzyme plays an unclear but crucial role in matrix mineralization[26].

Figure 4 clearly demonstrates a significant increase ($p < 0.01$, vs. vehicle control) in the level of cellular (ALP) activity of HOS 58 human osteoblastic osteosarcoma cells at LE concentrations from 3.25 µg/mL to 30 µg/mL. Higher concentrations of LE showed an even more pronounced increase in ALP activity but were considered to be far above permissible plasma levels (Data not shown). These data further support our hypothesis that LE acts mainly to expedite osteoblast maturation *in vitro*, as total cellular protein is not affected, while ALP activity (and thence ECM mineralization) is improved. The vehicle DMSO itself showed no increase of enzyme activity compared to cell control. The model substance *β*-glycerophosphate (2 mM) was used as a positive control and led to a significant elevation of ALP activity within the usual range indicating normal cell behavior.

**Figure 4.** The effect of *Lentinula edodes* total water extract on Alkaline Phosphatase activity of HOS 58 cells. A significant increase of ALP activity can be observed for 3–30 µg/mL. Each bar represents mean ± SD (n = 24) and was generated by three independent experiments. Difference at $p < 0.05$ was considered statistically significant. (**$p \leq 0.01$, *$p \leq 0.05$)

**Influence on Mineralization of Bone Cells.** To accomplish the testing of LE, we studied its effect on *in vitro* mineralization using Saos-2 cells. This delicate procedure is not fully understood and requires sophisticated ECM, an active ALP (in the preliminary stages), and a certain concentration of inorganic phosphorus. Mineralization as the last step of bone formation unambiguously shows the bone character of the cells. It is therefore used as an end-point measure for *in vitro* studies applying cells of osteoblastic lineage.

After 21 days of cultivation, Saos-2 cells grew in the absence of *β*-glycerophosphate (bGP) and did not show mineralization (Figure 5A). The addition of 2 mM bGP was recognized to be essential for the activation of the mineralization process and led to detectable calcium deposits of 10% of the total growth area (Figures 5B and 6). This observation had been made by others as well and also a bGP concentration up to 10 mM is frequently used to trigger mineralization[27].

**Figure 5.** Mineralized nodule formation by Saos-2 cells as demonstrated by Von Kossa staining after 3 weeks of culture. Without bGP (A), cells grew normally but did not mineralize. Cells cultured with bGP showed mineralization nodules (B), additionally. When co-stimulated with 20 µg/mL *Lentinula edodes* water extract (C), mineralized area was increased nearly 2 fold compared to bGP alone.

However, concentrations above 4 mM bGP were not tolerated by the cells in our experiment (data not shown). Number and size of mineralized nodules rose when Saos-2 cells were co-stimulated with 20 µg/mL (LE) total water extract and 2 mM bGP. In this case, area of mineralized nodules increased clearly from ~10% to ~22% (Figures 5C and 6). These results are in accordance with elevated ALP activity observed in HOS 58 cells and strongly assist the notion of osteogenic action of LE. A positive correlation between ALP activity and bone formation had been found by others, and it was suggested that greater ALP activity implies intensifying bone formation which can be observed in our results[28].

**Figure 6.** Influence of Lentinula edodes total water extract (20 µg/mL) on in vitro mineralization. Mineralization nodules (= Kossa positive objects) were quantified using CellExplorer 2001 software. Each bar represents mean ± SD (n = 10) and was generated by two independent experiments. Difference at $p > 0.05$ was considered statistically significant. (*$p \leq 0.05$).

## Experimental Section

**Chemicals and Biochemicals.** Cell culture plastic ware, fetal bovine serum (FBS), phosphate buffer (PBS), L-glutamine, trypsine and antibiotics were purchased from Biochrom KG (Berlin, Germany). Bovine serum albumin (fraction V), Iscove´s modification of Dulbecco´s medium (IMDM) with or without phenol red were purchased from Invitrogen (Karlsruhe, Germany). All other reagents were obtained from Sigma (Deisenhofen, Germany). HOS 58 cells were obtained as a gift from Professor A. Battmann (University of Giessen, Institute of Pathology, Germany). Saos-2 cells were purchased from DSZM (Braunschweig, Germany).

**Preparation of *Lentinula edodes* Extract.** Powdered material of dried *Lentinula edodes* (Berk.) Pegler fruit bodies were obtained from GAMU GmbH Krefeld, Germany. The powder was extracted with deionized water in a Soxhlet apparatus for 24 h. The extract was dried by lyophilization (yield: 11.5%) and stored at 4 °C. Before use, the extract was dissolved in DMSO (10 mg/mL) and sterilized by filtration.

**HPLC Fingerprint.** *L. edodes* extract was dissolved in water (3 mg/mL) and 10 µL were injected on a column (Merck Lichrospere, 5 µm, RP18). Analysis was performed on a Shimadzu LC2010AHT (Kyoto, Japan). Mobile phase was water – 0.1% formic acid (A) and methanol (Fisher Scientific, gradient grade) – 0.1% formic acid (B) for 15 min as a linear gradient (10% B to 15% B) with a flow rate of 0.8 mL/min. Signals were detected using a UV detector using 206 nm and 254 nm.

**Cell Culture.** Human osteosarcoma cells HOS 58, and Saos-2 were grown as monolayer in IMDM with 10% fetal bovine serum, 2 mM L-glutamine, and 1% penicillin-streptomycin solution (penicillin 10000 IE/mL; streptomycin 10000 µg/mL). Both cell lines were grown at 37 °C in 95% air humidity, and 5% $CO_2$, and sub-cultured routinely.

For assays, HOS 58 cells were grown to confluence in 96-well plates for 48 h. After washing with PBS twice, the medium was changed to IMDM without phenol red supplemented with 0.05% bovine serum albumin, 2 mM L-glutamine, and 1% antibiotics (assay medium). Different concentrations of (LE) extract in assay medium were prepared using a stock (10 mg/mL in DMSO) and serial dilution with the medium. Final DMSO concentration did not exceed 0.05%. Further procedures are indicated below.

**Cell Vitality and Cell Proliferation Assay (MTT Assay):** The MTT assay was used to measure cell proliferation rate and cell viability. HOS 58 cells (see cell culture) were incubated with different concentrations of LE (0.8 µg/mL–125 µg/mL) for 43 h. After washing, 20 µL MTT in IMDM (5 mg/mL, Sigma, Deisenhofen, Germany) were added to each well. Plates were incubated at 37 °C in 95% air humidity, and 5% $CO_2$ for a further 5 h. At the end, MTT solution was removed and crystals dissolved in 200 µL DMSO. After mixing thoroughly, plates were incubated for 5 min and absorbance was also measured at 590 nm. Cell viability was calculated as a percent of the vehicle control[29,30].

**Cell Maturation Assay.** The cell maturation assay was used to determine the osteoblasticity of osteoblastic cells. HOS 58 cells (see cell culture) were incubated with different concentrations of GF for 5 days. Medium was exchanged on day 3. Cells then were lysed and enzyme activity of ALP was determined in the supernatant.

**Cell Lysis.** Cells treated with LE extracts or vehicle were washed with PBS and disrupted by adding 100 µL of 0.1% Triton X-100 in 0.1 M Tris-HCl, pH 9.8 (lysis buffer) followed by freeze/thawing and vigorous mixing. The obtained suspension was centrifuged and the supernatant (cell lysate) assayed for protein content and ALP activity.

**Protein Quantification.** Total cellular protein was determined by using Roti-Nanoquant reagent (Roth GmbH, Karlsruhe, Germany); a modified Bradford method; according to the manufacturer's instructions[31]. Briefly, 10 µL cell lysate was diluted with PBS (1:4) in a microtiter plate. Roti-Nanoquant (200 µL) reagent was added, mixed and the O.D. was recorded at 405 and 620 nm (anthos labtec, Salzburg, Austria). Total protein content was calculated from a standard curve using bovine serum albumin (BSA).

**Alkaline Phosphatase Activity.** Cellular ALP activity was determined by the release of 4-nitrophenol (4-NP) from 4-nitrophenyl phosphate (4-NPP). An aliquot of cell lysate was mixed with 0.2 M aminopropanol buffer pH 9.8 (AMP) and 24 mM (4-NPP) in (AMP). After incubation (37 °C) reaction was stopped by 0.5 M NaOH (50 µL) and O.D. was read out at 405 nm. Concentration of 4-NP was calculated utilizing a calibration curve.

**Mineralization Assay.** Saos-2 cells were seeded in 24 well-multiwell plates ($10^4$ cell/well) using growth medium (see cell culture) and grown to 90% confluence. Medium was discarded and cells maintained in the assay medium (see cell culture) with or without the LE extract (20 µg/mL) for 21 days. The medium was changed every 2 days. Mineralization was triggered by adding 2 mM $\beta$-glycerophosphate (bGP) to medium continuously. At the end of incubation time, cells were stained for mineral deposition by using the Von Kossa method[32]. After washing with warm PBS, the cells were then fixed with 5% glutardialdehyde (Grade II, Sigma, Deisenhofen, Germany) in deionized water for 30 min. Cell layers were washed twice with deionized water and incubated with 5% silver nitrate in water under UV radiation for 50 min. Then cells were washed 3 times with deionized water, and 5% sodium thiosulphate in water was added for 2 min. After last washing step, cells were examined for stained (black) spots under microscope and photographed utilizing a Canon EOS 20 D digital camera. Changes in both the number of mineralization nodules and its areas were quantified using CellExplorer 2001 software (BioSciTec, Frankfurt, Germany).

**Statistical Analysis.** For ALP determination, three independent experiments were carried out with 8 replicates each and expressed as mean ± SD. Statistical differences were analyzed using single side ANOVA: p-values $\leq 0.05$ were considered significant. In case of mineralization assay each bar represents mean ± SD of ten pictures from two independent experiments.

## Conclusions

In this study a water extract of the mushroom *Lentinula edodes* was found to stimulate mineralization and alkaline phosphatase activity of human osteoblastic cell cultures for the first time. Utilizing pharmacologically relevant concentrations (< 30 µg/mL), our data show that the extract enhances osteogenicity of cultured bone cells.

This positive effect on bone cells with comparatively low doses of extract suggests the potential existence of active compounds stimulating bone formation and bone mineralization, which remains to be identified in future. The calcium content of *L. edodes* is not remarkably high[33,34] so that a contribution of calcium to the observed effects is unlikely.

In conclusion, it has been demonstrated that aqueous extract of *L. edodes* has a bone enhancing effect *in vitro*, suggesting a possible role in the prevention of osteoporosis. LE extract could thus help to delay late complications of diabetes; especially diabetic bone disorders. This needs to be further evaluated in animal models and human double blind placebo clinical randomized trials to support the proposed conclusions.

## Acknowledgments

We thank Monika Matthias and Simon Merdivan, Institute of Pharmacy, Greifswald, for preparation and chromatographic investigation of the extract of *Lentinula edodes.*

**Open Access** This article is distributed under the terms of the Creative Commons Attribution License which permits any use, distribution, and reproduction in any medium, provided the original author(s) and source are credited.
